# Factors associated with early initiation of breastfeeding among children less than 24 months old: the 2019 Ethiopian mini demographic and health survey

**DOI:** 10.1186/s13690-022-00920-4

**Published:** 2022-07-06

**Authors:** Tadele Abate Lucha, Admassu Ketsela Mengistu

**Affiliations:** 1 Department of Neonatal Nursing, Menelik II Medical & Health Sciences College, Kotebe Metropolitan University, Addis Ababa, Ethiopia; 2 Department of Pharmacy, Menelik II Medical & Health Sciences College, Kotebe Metropolitan University, Addis Ababa, Ethiopia

**Keywords:** Breastfeeding, Early initiation of breastfeeding, Ethiopia, Mother

## Abstract

**Background:**

The world Health Organization recommended that all mothers be supported to initiate breastfeeding as soon as possible after birth, within the first hour. This study examined the determinants of early initiation of breastfeeding in Ethiopia by using data from the 2019 Ethiopia Mini Demographic and Health Survey.

**Methods:**

The data for this study was extracted from the 2019 Ethiopia Mini Demographic and Health Survey. A total of 1948 children aged less than 24 months at the time of the survey were included for analysis from the nine regional states and two city administrations. The analysis was carried out in STATA Version 14.2 software using survey commands to account for the complex sample design and apply sampling weights. A manual backward stepwise elimination approach was applied.

**Result:**

The prevalence of early initiation of breastfeeding in Ethiopia was noted to be 75.2% [95% CI (71.9, 78.5%)]. In the multivariable analysis, mothers who had vaginal deliveries [AOR = 3.02 (95% CI: 1.55, 5.88)] had 3 times higher odds practicing early initiation of breastfeeding compared to mothers who had a cesarian section. Mothers aged between 35 and 49 years [AOR = 2.40, 95% CI: 1.20, 4.49] had 2.4 times higher odds of practicing early initiation of breastfeeding compared to mothers aged below 20 years. In addition, early initiation of breastfeeding was also associated with the region where mothers resided, in particular mothers in Amhara and Somali region, had lower odds of practicing early initiation of breastfeeding as compared with mothers residing in Tigray region.

**Conclusion:**

Early initiation of breastfeeding in Ethiopia was found to be significantly associated with mode of delivery, mother’s age, and region. As a result, raising awareness about early initiation of breastfeeding is especially important for mothers who have had a cesarean section, which could be accomplished with the help of the health extension workforce.

## Background

According to the world health organization (WHO) breastfeeding indicator, early initiation of breastfeeding is defined as the percentage of children born in the last 24 months who were put to the breast within 1 h after birth [1]. WHO recommends that all mothers should be supported to initiate breastfeeding as soon as possible after birth, specifically within the first hour [2].

Evidence indicates Early initiation of breastfeeding (EIBF) decreases the risk of infection, neonatal mortality, and underweight [3, 4]. EIBF is associated with a 64% reduction in the incidence of non-specific gastrointestinal tract infections and otitis media by 23%. It also has short and long-term health benefits for the mother by decreasing postpartum blood loss and rapid involution of the uterus [5]. A study from Indonesia has documented that 22% of neonatal deaths could have been prevented if all neonates were put on breastfeeding within the first hour and around 16% of neonatal deaths would have been prevented if all neonates were breastfed from the first day [6]. In India, 15% of all causes of neonatal mortality could have been prevented if all babies were exposed to early breastfeeding [7]. On the other hand, children who didn’t get an early initiation of breastfeeding were more likely to be stunted than those who were breastfed early [8]. The reason behind this is the fact that early milk is enriched in antibiotics, immune and growth factors that help to ensure infant survival [9].

Though the importance of EIBF is recognized widely, the weighted global prevalence of early initiation of breastfeeding for the years between 2010 and 2018 was only 51.9% [10]. As the WHO infant and young child feeding indicator on EIBF practice shows, approximately 40% of mothers in Bangladesh, Haiti, and Uganda early initiate of breastfeeding their youngest child within 1 h of birth. More than half of mothers reported EIBF in Kenya and Zambia (58 and 56%, respectively), and this percentage reached 67% in Ethiopia and Zimbabwe. Only 23% of mothers reported initiating BF early in India [4]. Research indicates that the trends and prevalence of EIBF in Ethiopia was 51% in 2000, 69% in 2005, 52% in 2011, and 74.3% in 2016 [11, 12].

Despite improvement in the prevalence of EIBF from 51% in 2000 to 74.3% in 2016, this percentage is still low as compared to the EIBF target of 92% by the end of the year 2020 set by the Ethiopian ministry of health [13].

Although there are a number of studies investigating the factors associated with the early initiation of breastfeeding in Ethiopia, the majority of them are conducted in a small town or district, making the clustering effect difficult to account for. Therefore, this study uses a complex survey design to account for the clustering effect.

The current study aimed to use the 2019 EMDHS to determine factors that determine EIBF in mothers of infants aged less than 24 months. This may help to evaluate the Ethiopian ministry of Health’s health sector transformation plan (HSTP), which targets a decrease in neonatal mortality rate from 28 to 10% and stunting from 40 to 26% by the end of 2020 [14].

## Methods

### Study area and data source

The Ethiopia Mini Demographic and Health Survey 2019 (EDHS) is a community-based cross-sectional study conducted from March 21, 2019, to June 28, 2019. The study was conducted in Ethiopia (3^o^-14^o^ N and 33^o^ - 48°E), situated at the eastern horn of Africa. The country covers 1.1 million square kilometers and has a great geographical diversity, which ranges 4550 m above sea level down to the Afar depression to 110 m below sea level.

The data for this study was extracted from the 2019 EMDHS [15]. The 2019 EMDHS is the second EMDHS and the fifth DHS implemented in Ethiopia. The survey was conducted in nine regional states and two city administrations in the Federal Democratic Republic of Ethiopia [15].

### Sampling procedure

The 2019 EMDHS sample was stratified and selected in two stages. Each region was stratified into urban and rural areas, yielding 21 sampling strata. In the first stage, a total of 305 enumeration areas (93 in urban areas and 212 in rural areas) were selected with a probability proportional to enumeration area size based on the 2019 Ethiopian Population and Housing Census (EPHC) frame and with independent selection in each sampling stratum [15]. In the second stage of selection, a fixed number of 30 households per cluster were selected with an equal probability of systematic selection from the newly created household listing. All women aged 15–49 who were either permanent residents of the selected households or visitors who slept in the households on the night before the survey were eligible to be interviewed [15].

### Data collection

The survey was conducted based on a nationally representative sample that provided estimates at the national and regional levels as well as for urban and rural areas. The survey was conducted in 9150 residential households (2790 in urban areas and 6360 in rural areas). The sample was expected to result in about 7959 completed interviews with women aged 15–49 years (2636 in urban areas and 5323 in rural areas) [15]. Our analysis only included children less than 24 months of age living with an eligible respondent, in accordance with the denominator of the EIBF definition, which resulted in a total weighted sample of 1948.

### Outcome variable

We used EIBF as the outcome variable, using the recommended definition of children born in the last 24 months who were put to the breast within 1 h [1]. The outcome variable was measured as 1 if a mother-initiated breastfeeding in the first hour after birth and 0 if delayed. This indicator was self-reported by the mother.

### Independent variable

The independent variables were mother’s education, maternal age, wealth index of household, religion, place of residence, region, professional antenatal and postnatal care, place of delivery, mode of delivery, type of birth, sex of child, marital status of the mother, and parity, which were selected from the available similar studies on the subject [11, 16–19]. The EDHS use five wealth quintiles. To determine household level wealth index households are given scores based on the number and kinds of consumer goods they own, ranging from a television to a bicycle or car, and housing characteristics such as source of drinking water, toilet facilities, and flooring materials. These scores are derived using principal component analysis. National wealth quintiles are compiled by assigning the household score to each usual (de jure) household member, ranking each person in the household population by her or his score, and then dividing the distribution into five equal categories, each comprising 20% of the population [15].

### Statistical analysis

The analysis was carried out in STATA Version 14.2 software using survey commands to account for the complex sample design and apply sampling weights. Sample weights were applied to account for the variable probability of study participants being selected based on their residency region. The “Svy” command were used to make changes to the cluster sampling design, weights, and standard error calculation. To account for the complex sampling strategy and weights, survey logistic regression was used. Using binary logistic regression analysis, the relationship between the independent variable and vitamin A supplementation was first examined. Second, the factors associated with vitamin A supplementation (*p* value < 0.25) were investigated using a multivariate logistic regression model. We used a manual backward stepwise elimination approach by ordering the covariates from most to least important. With 95% confidence intervals, both unadjusted and adjusted odds ratios (OR) were presented (95% CI). Statistical significance was declared at the level of *p* value < 0.05.

## Result

### Bassline characteristics

The prevalence of EIBF in Ethiopia was noted to be 75.2 95% CI (71.9, 78.5%). Table 1summarizes the findings. The principal percentage of children lived in rural areas (72.2%), mainly in the regions of Oromia (39.1%), Amhara (21%), and SNNP (19.4%). One-third of the mothers (33.2%) had only a primary education.

Concerning mothers’ age, overall, 73.5% were between 20 and 34 years old. Most of the mothers (95.2%) described themselves as currently married at the time of the survey. In terms of place of delivery, 55.3% of mothers delivered at a health facility, and of these, 6.4% delivered by cesarean section. Regarding the number of antenatal visits, about 30.3% of mothers reported having made at least 1–3 antenatal clinic visits and 45.3% had made more than 4 visits during pregnancy. Only 13% of babies had postnatal checkups (Table 1).

**Table 1:** Background characteristics of mother and child < 24 month, Ethiopia Mini Demographic and Health Survey 2019 (*n* = 1948)

Variable	Frequency (n)	Percent (%)
**Region**		
Tigray	145	7.4
Afar	28	1.5
Amhara	409	21.0
Oromia	761	39.1
Somali	118	6.1
Benishangul	23	1.2
SNNPR	377	19.4
Gambela	10	0.5
Harari	5	0.3
Addis Ababa	60	3.1
Dire Dawa	11	0.6
**Place of residence**		
Urban	530	27.2
Rural	1418	72.8
**Maternal Educational Level**		
No education	882	45.3
Primary	791	40.6
Secondary/higher	275	14.1
**Religion**		
Orthodox	709	36.4
Protestant	526	27.0
Muslim	687	35.3
Traditional	25	1.3
**Wealth index**		
Poorest	420	21.5
Poorer	414	21.2
Middle	364	18.7
Richer	336	17.3
Richest	414	21.3
**Types of birth**		
Single birth	1929	99.0
Twine	19	1.0
**Child sex**		
Male	978	50.2
Female	970	49.8
**Place of delivery**		
Home	872	44.7
Health	1076	55.3
**Antenatal care**		
No ANC visit	474	24.3
1–3	591	30.3
4+	883	45.3
**Post-natal check up**		
No	1695	87.0
Yes	252	13.0
**Age of Mother**		
< 20	160	8.2
20–34	1431	73.5
35–49	357	18.3
**Parity**		
1–2	865	44.4
3–4	490	25.1
5–6	337	17.3
7+	256	13.2
**Current marital status of mother**		
Married	1853	95.2
No longer living together/separated	94	4.8
**Mode of delivery**		
Normal (vaginal)	1824	93.6
Cesarean section	124	6.4

### Factors associated with early initiation of breastfeeding in Ethiopia

Mothers who had deliveries by vaginal method [AOR = 3.02, 95% CI: 1.55, 5.88] had 3 times higher odds of practicing early initiation of breastfeeding compared with cesarian section delivery. In terms of mothers’ age, those mothers aged between 35 and 49 years [AOR = 2.40, 95% CI: 1.20, 4.49] 2.4 times higher odds of practicing early initiation of breastfeeding when compared with mothers aged below 20 years (Table 2).

**Table 2:** Factors associated with early initiation of breastfeeding in Ethiopia, Ethiopia Mini Demographic and Health Survey 2019

Variables	Unadjusted OR, [95%CI]	Adjusted OR, [95%CI]
**Mode of delivery**		
Normal	2.89 [1.51, 5.54]	3.02 [1.55, 5.88] ***
Cesarean section	1	
**Postnatal check up**		
No	1	
Yes	0.76 [0.49, 1.16]	
**Parity**		
1–2	1	
3–4	1.06 [0.75, 1.49]	
5–6	1.30 [0.89, 1.90]	
7+	2.01 [1.18, 3.41]	
**Age of Mother**		
< 20	1	**1**
20–34	1.66 [0.91, 3.04]	1.88 [0.96, 3.68]
35–49	2.20 [1.30, 3.73]	2.40 [1.20, 4.79] *
**Age of child (months)**		
0–5	1	
6–11	0.81 [0.48, 1.37]	
12–17	0.60 [0.35, 1.03]	
18–23	1.13 [0.68, 1.87]	
**Region**		
Tigray	1	1
Afar	0.67 [0.36, 1.26]	0.64 [0.34, 1.23]
Amhara	0.55 [0.31, 0.97]	0.53 [0.30, 0.93] *
Oromia	1.70 [1.00, 2.90]	1.58 [0.91, 2.76]
Somali	0.48 [0.25, 0.92]	0.40 [0.20, 0.79] **
Benishangul	1.09 [0.55, 2.16]	1.13 [0.50, 2.55]
SNNPR	0.86 [0.53, 1.38]	0.82 [0.50, 1.36]
Gambela	1.31 [0.67, 2.57]	1.24 [0.57, 2.68]
Harari	0.97 [0.52, 1.78]	0.94 [0.50, 1.77]
Addis Ababa	0.90 [0.45, 1.78]	1.07 [0.50, 2.32]
Dire Dawa	0.99 [0.56, 1.73]	1.77 [0.65, 2.09]

* *p* value < 0.05

**p value < 0.01

**p value < 0.001

In addition, early initiation of breastfeeding was also associated with regions where mothers resided, particularly in regions such as Amhara and Somali, which had lower odds of practicing early initiation breastfeeding as compared with mothers residing in the Tigray region (Table 2).

## Discussion

In this study, we investigated the determinants of early initiation of breastfeeding among women aged 15–49 years in Ethiopia using secondary data from the EMDHS 2019. The overall prevalence of early initiation of breastfeeding was 75.2% [95% CI (71.9, 78.5%)]. According to the WHO classification, the reported EIBF prevalence in this study is categorized as good [20]. It is still lower when compared to the previous studies of EIBF prevalence in other countries, such as Angola (98.4%), Cuba (89.2%) and Sri Lanka (85.5%) [21], and also lower than the national 92% EIBF targeted by the health sector development program of Ethiopia [13]. However, the overall prevalence of EIBF in Ethiopia is much higher compared to the economic community of West Africa state (ECOWAS) (43%) [22], Ghana (55.7%) [17], Tanzania (71.4%) [23], the kingdom of Saudi Arabia (43.6%) [24], India (41.5%) [25], Indonesia (57%) [26] and the Middle East (34.3%) [27]. The reason for the higher prevalence of EIBF in the current study compared to the previous study might be due to the successful health extension program implementation in the Ethiopian primary health care system. The health extension workers identify pregnant mothers and refer them to the nearby health facilities for delivery. This might enable the mothers to obtain information on the importance of EIBF from health care providers [28]. Since the last EDHS in 2016, there has been no development in EIBF in Ethiopia, which could be explained in part by the country’s turbulent political situation, which has caused the government to declare a state of emergency, which could have had a detrimental influence on health services.

Mode of delivery was significantly associated with the early initiation of breastfeeding in the present study. A mother who delivered by vaginal delivery was around 3 times more likely to initiate breastfeeding within 1 h than those who gave birth via cesarean section. This finding is supported by a similar study done in Ethiopia, a secondary analysis of EDHS 2016 [11, 16], different regions in Ethiopia [29–32], and different countries such as Saudi Arabia [25], Sudan [33], Tanzania [34, 35], Uganda [36, 37], Ghana [17], Nigeria [18], Namibia [38], West Africa state [23], Nepal [39, 40], India [26], Indonesia [8, 27, 41], Bangladesh [42, 43], South Asia [44], Middle East [28], Turkey [45], Romania [46], and a secondary analysis paper of the WHO Global survey published in 2017 also showed EIBF to be significantly lower among women with caesarean section delivery [22]. This may be due to the procedure taking longer, pain after the procedure, effects of anesthesia and tiredness that make it difficult to initiate breastfeeding early and the time of postoperative care, which delays mother-baby contact [45]. As a result, health professionals and pregnant mothers could be informed about the detrimental link between a caesarean delivery and breastfeeding, as well as the influence on infant well-being.

In terms of mothers’ age, those mothers aged between 35 and 49 had 2.4 times higher odds of practicing early initiation of breastfeeding when compared with mothers aged below 20 years. This finding is supported by a systematic literature review in South Asia [45].

There was variation between the levels of early initiation of breastfeeding among the administrative regions of Ethiopia, which could be explained by differences in health-care utilization, culture, and socioeconomic status across the different regions of Ethiopia.

One of the study’s strengths is that we used data from the 2019 EMDHS, which is a national survey. The major limitation the current study is that it is subject to recall bias. The other weakness was that some important possible factors that could affect the practice like birth order, mother’s occupation, and father’s characteristics were not included due to incompleteness of information.

## Conclusion

EIBF was found to be significantly associated with mode of delivery, mothers age and region of residence of the mothers. Therefore, the authors recommend increasing awareness about EIBF, especially for mothers delivering by cesarian section and younger mothers, which could be done utilizing the health extension workforce throughout the Nation.

## Data Availability

The survey datasets used in this study was based on publicly available dataset that is freely available online with no participant’s identity from http://www.dhsprogram.com/data/available-datasets.cfm. The minimal data used for this study are available from the corresponding author on reasonable request.

## Abbreviations

AOR: Adjusted Odds Ratio
CI: Confidence Interval
COR: Crude Odds Ratio
EIBF: Early Initiation of Breastfeeding
EMDHS: Ethiopian Mini Demographic and Health Survey
EPHC: Ethiopian Population and Housing Census
HSTP: Health sector transformation plan
SNNP: Southern Nations Nationalities and Peoples
WHO: World health organization
